# The Complete Mitochondrial Genome of *Dendrogale murina* (Tupaiidae) and Phylogeny of Scandentia

**DOI:** 10.3390/genes14030624

**Published:** 2023-03-01

**Authors:** Tatyana Petrova, Olga Bondareva, Semyon Bodrov, Alexei Abramov, Natalia Abramson

**Affiliations:** 1 Zoological Institute RAS, 199034 Saint-Petersburg, Russia; 2 Joint Vietnamese-Russian Tropical Research and Technological Centre, Nguyen Van Huyen, Nghia Do, Cau Giay, Hanoi 650000, Vietnam

**Keywords:** mitochondrial genome, phylogeny, divergence dating, Scandentia, *Dendrogale murina*, *Ptilocercus lowii*, Vietnam

## Abstract

In this paper, we report the complete mitochondrial genome of the northern smooth-tailed treeshrew *Dendrogale murina*, which was sequenced for the first time using the Illumina next-generation sequencing (NGS) technology. The total length of the mitochondrial genome is 16,844–16,850 bp and encodes 37 genes, including two ribosomal RNAs (rRNAs) *12S* and *16S*, 22 transfer RNAs (tRNAs), 13 protein-coding genes (PCGs), and a D-loop in the characteristic arrangement of family Tupaiidae (Mammalia: Scandentia). The overall base composition of the complete mitochondrial DNA is A (33.5%), C (25.5%), G (13.9%), and T (27.1%). Phylogenetic analysis of Scandentia mitochondrial genomes showed a classic pattern, which was revealed previously while using individual phylogenetic markers. The result of the current study is consistent with one based on the latest morphological studies, with the basal position of *Ptilocercus* and *Dendrogale* sister to the rest of the Tupaiidae genera. The divergence time of the *Dendrogale* genus is estimated as Eocene–Oligocene, with the mean value of 35.8 MYA, and the *Ptilocercus* genus probably separated at about 46.3 MYA. We observe an increase in the age of all nodes within the Scandentia, except for a decrease in the age of separation of *Ptilocercus.* This result can be explained both by the addition of new mitochondrial genome data in the analysis and the usage of new calibration points from recently published data.

## 1. Introduction

The order Scandentia (treeshrews), for a long time, has been the focus of evolutionary and phylogenetic studies with emphasis on the position of the order among other eutherian mammals and on the origin of primates. These studies were performed using both morphological and molecular tools [1,2,3,4,5]. Usually in these comparative studies, one or two species of the genus *Tupaia* Raffles, 1821, were used as a typical representative of the order, although the diversity of the order Scandentia is not limited to the family Tupaiidae Gray, 1825. Thus, until recently, the interrelationships of taxa within the order received less attention. Meanwhile, the various studies with the use of both molecular and morphological analyses performed in the first decade of the 21th century showed the high heterogeneity of taxa within the order [6,7,8,9,10,11]. Hence, the evolutionary history and phylogenetic relationships of the taxa within this very ancient and enigmatic group, the recent species of which represent only a small portion of past diversity, largely remained obscure. According to the latest systematic account [12], the order is represented with two families: Tupaiidae and Ptilocercidae Lyon, 1913. The latter is monotypic and includes the only genus, *Ptilocercus* Gray, 1848, with one species, *Ptilocercus lowii* Gray, 1848, distributed in the western part of Southeast Asia [12]. The latter species is remarkable, being a “living fossil”, as it still retains many plesiomorphic morphological features very similar to the extinct representative of this genus, *Ptilocercus kylin* Li and Ni, 2016, aged as the early Oligocene at 34 MYA [13]. Family Tupaiidae unites the majority of order representatives and includes four genera: *Tupaia* and *Anathana* Lyon, 1913; *Dendrogale* Gray, 1848; and *Urogale* Mearns, 1905. The most speciose, widespread, and best-studied is the *Tupaia*, which includes not less than 15 species, while genera *Anathana* (Madras treeshrews) and *Urogale* (Mindanao treeshrews) are monotypic and narrow-ranged, inhabiting the southern part of the Peninsular India and southern Philippines, respectively.

The genus *Dendrogale* is represented by two species: northern smooth-tailed treeshrew *D. murina* Schlegel and Müller, 1843, and Bornean smooth-tailed treeshrew *D. melanura* Thomas, 1892, both of which are extremely poorly studied species. *D. murina* is distributed from Eastern Thailand through Cambodia and southeast Laos to Southern and Central Vietnam [14,15]. The species everywhere is rare or is highly localized in occurrences that are reflected in the scarcity of both ecological and genetic information on the species. Limited biological sampling of the species might be interpreted as evidence of genuine rarity [14]. Hawkins [16] and Francis [17] summarized some data on the ecology and natural history of *D. murina*, whereas the genetic data is still scarce.

The position of genus *Dendrogale* within the family Tupaiidae was long debatable. Butler [18], on the basis of thorough analysis of dental features of all Scandentia species, suggested one of several possible interpretations with respect to the position of *Dendrogale*, which he, as well as Steele [19], chose to depict as nested within the subfamily Tupaiidae rather than as its basal-most member. Later on, reassessment of all characters previously considered in morphological studies [10,20] showed the sister position of *Dendrogale* to be the clade uniting all other treeshrew species and the basal position of *Ptilocercus* within the order. This hypothesis was later supported by a few molecular studies [11,21]. Phylogeny of Scandentia was reconstructed using mitochondrial *12S* and *16S rRNA* genes and several nuclear genes [11,21,22].

Complete mitochondrial genomes have been effectively used to understand the evolutionary relationships among numerous taxa of vertebrate and invertebrate animals. The mitochondrial genomes provided robust phylogenetic signals that were much more informative than the signals obtained using single mitochondrial genes. Several seminal studies have proven that phylogenetic reconstructions based on complete mitochondrial genomes showed better resolution than the trees based upon a few single loci [23,24,25]. Meanwhile, the genetic information and structural motifs of the *Dendrogale* mitochondrial genome are still unknown. The complete mitochondrial genomes based on phylogenetic reconstruction [26] analyzed only the *Tupaia* species and did not include both the basal Scandentia genera *Dendrogale* and *Ptilocercus.* To fill this gap of knowledge, the present study first aims to determine the complete mitochondrial genome of *Dendrogale murina* from Southern Vietnam. Second, we aim to test which alternative hypothesis of the *Dendrogale* position is supported by mitochondrial genome-based phylogeny. To this end, we additionally partly assemble the mitochondrial genome of the pen-tailed treeshrew *P. lowii* (from the previously published Sequence Read Archive (SRA) datasets and partial mitochondrial cytochrome *b* gene) and conduct phylogenetic analyses and divergence time estimation to infer the evolutionary relationship of the genera *Dendrogale*, *Tupaia,* and *Ptilocercus* within the order Scandentia.

## 2. Materials and Methods

### 2.1. Material

**Figure 1:**
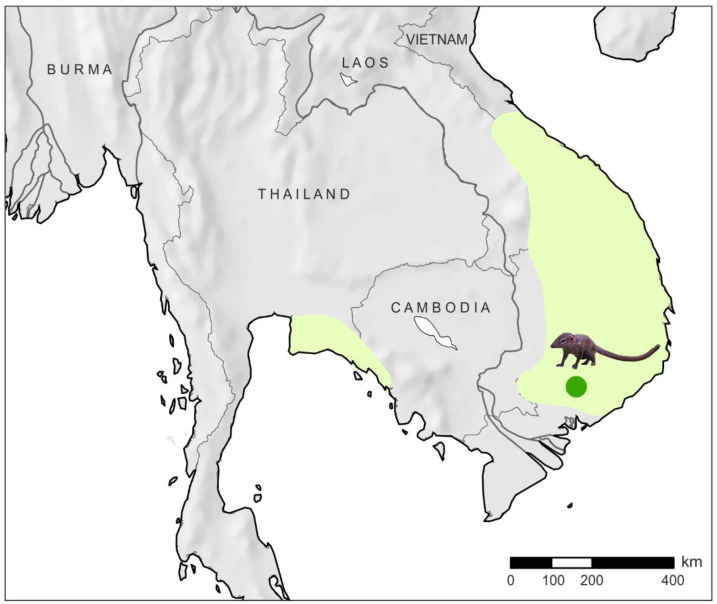
*Dendrogale murina* distribution range and collection locality. Distribution range is given according to Timmins [15].

Two specimens of *Dendrogale murina* were collected in Southern Vietnam, Dong Nai Province, Vinh Cuu District, 6 km north of Ba Hao Village, 11.309° N, 107.079° E (Figure 1). Muscle tissues were fixed in 96% ethanol. Voucher specimens were deposited under the numbers ZIN 100298 and ZIN 100299 in the collection of the Laboratory of Theriology of the Zoological Institute of Russian Academy of Sciences (ZIN), Saint Petersburg, Russia; the tissue and DNA of the specimens were deposited under the numbers 5842 and 5843 in the tissue collection of the Laboratory of Evolutionary Genomics and Paleogenomics of the ZIN. In addition, data were downloaded from the NCBI SRA and Nucleotide databases (https://www.ncbi.nlm.nih.gov/, accessed on 20 June 2022): *Tupaia belangeri* Wagner, 1841 (NC_002521); *T. minor* Günther, 1876 (MT442050); *T. montana* Thomas, 1892 (NC_054191); *T. nicobarica* Zelebor, 1869 (MW751815); *T. splendidula* Gray, 1865 (NC_050994); *T. tana* Raffles, 1821 (NC_050992); *P. lowii* (SRR2577514, SRR6053052, and MK111982); and *Galeopterus variegatus* Audebert, 1799 (JN800721).

### 2.2. DNA Extraction, Library Preparation, and Sequencing

Total genomic DNA was isolated from the tissue fragment dissected from the femoral muscle. Homogenization was performed using the mill TissueLyser LT (Qiagen, Hilden, Germany) according to the original protocol for animal and human tissues. Genomic DNA was extracted using the DNeasy Blood & Tissue Kit (Qiagen, Hilden, Germany) according to the original protocol and stored at −20 °C. 

NGS libraries were prepared using the NEBNext Ultra II DNA Library Prep Kit for Illumina (New England Biolabs, Beijing, China). The resulting PCR products were purified and concentrated using AMPure XP beads (Beckman Coulter, Beverly, MA, USA). The concentration of samples was measured using a Qubit 4 fluorometer (Thermo Fisher Scientific, Waltham, MA, USA), while the final quality control of the libraries was implemented using the Bioanalyzer 2100 instrument and the DNA High Sensitivity Kit (Agilent, Boulder, CO, USA).

Sequencing was performed on an Illumina HiSeq 4000 system, resulting in raw pair-end reads of 75 bp. DNA quality was checked with a Qubit 4 fluorometer, and the final distribution of lengths of the libraries’ adapter content checking was conducted using Bioanalyzer 2100 (Agilent, Boulder, CO, USA). DNA extraction, library preparation, and sequencing were performed in the Core Sequencing Centre of Kurchatov Centre for Genome Research (National Research Centre “Kurchatov Institute”, Russia).

### 2.3. Mitochondrial Genome Assembly, Annotation, and Sequence Analyses

The quality of raw reads was evaluated using FastQC ver. 0.11.9 [27], reads were cleaned from Illumina adapters, and overrepresented sequences and low-quality reads (< Q20) were cleaned using the Trimmomatic v0.39 [28] and FASTP software [29]. Two mitochondrial genomes were assembled from clean reads of two *D. murina* specimens using plasmidSPAdes version 3.10.1 [30,31]. *P. lowii* mitochondrial genome assembly was conducted by mapping to a reference from a consensus mitochondrial genome of six *Tupaia* species in Geneious Prime 2019.1 (Biomatters Ltd., Auckland, New Zealand, https://www.geneious.com, accessed on 26 November 2021) using the Geneious mapper with default settings. Fragments with a minimum of 10× coverage were used to generate the consensus sequence. The partially assembled sequence of mitochondrial cytochrome *b* gene (mt *CYTB*) was verified and complemented with the *CYTB* sequence downloaded from the NCBI Nucleotide database (MK111982).

The assembled mitochondrial genomes were annotated using the MITOS web server [32] with the default settings. Gene boundaries were checked and refined by alignment against published mitochondrial genome sequences of the *Tupaia* species. 

Nucleotide composition and codon usage were calculated using Geneious Prime 2019.1 (Biomatters Ltd., Auckland, New Zealand). To calculate the base composition skew, we used previously known formulas: AT skew = (A − T) / (A + T) and GC skew = (G − C) / (G + C) [33]. Both analyses were calculated using full-length mitochondrial genomes.

### 2.4. Phylogenetic Reconstruction and Divergence Time Estimation

Phylogenetic reconstruction of order Scandentia was conducted based on the alignment of complete mitochondrial genomes of six *Tupaia* species, two complete mitochondrial genomes of *D. murina*, and a partial mitochondrial genome of *P. lowii.* The Sunda flying lemur *G. variegatus* was used as an outgroup. The D-loop fragment was excluded from the alignment due to its high variability. In total, 36 genes (13 PCGs, two rRNAs, and 22 tRNAs) were included in the phylogenetic reconstruction. Mitochondrial genomes (except D-loop) were aligned with Mauve 1.1.1 [34] implemented as a plugin for Geneious Prime 2019.1.

The best-fit model (GTR + F + I + G4) was estimated in ModelFinder [35] using the Bayesian information criterion (BIC). The maximum-likelihood (ML) tree was constructed using the IQ-TREE web server http://iqtree.cibiv.univie.ac.at/ [36] with 1000 bootstrap support.

The estimation of divergence times among Scandentia species was calculated using the Bayesian relaxed-clock method in the BEAST v2.6.7 software [37]. The GTR + I + G substitution model, empirical base frequencies, and relaxed uncorrelated log-normal clock with the Yule speciation model was applied as Tree prior. Three fossil calibration points were applied to constrain the analysis. The total tree height (node A) was considered a log-normal prior (with the offset = 65.79 MYA and 95 % HPD of 126-67 MYA), as was done in Vries and Beck [38], according to the fossil *Purgatorius mckeeveri* Wilson Mantilla et al., 2021, from the early Palaeocene [39]. The most recent common ancestor (MRCA) of Scandentia representatives (node B) was calibrated with a log-normal distribution (with the offset = 34 MYA and 95 % HPD of 65-34 MYA), based on the fossil of *P. kylin* from the early Oligocene [13]. The MRCA of *Tupaia* (node C) was calibrated with a log-normal distribution (with the offset = 18 MYA and 95 % HPD of 28-18 MYA), based on the fossil of *Tupaia miocenica* Mein and Ginsburg, 1997 [40]. 

Two replicate runs of 100 million Markov chain Monte Carlo (MCMC) generations each were performed, sampling trees and parameter estimators every 1000 generations. The convergence of run parameters was examined using the effective-sample-size (ESS) statistics in Tracer v1.7 [41]. To combine these runs, log files were summarized in LogCombiner of the BEAST package, and the first 25 million generations were discarded as burn-in. The final time tree was summarized using TreeAnnotator v2.6.7, using the maximum clade credibility tree option and fixing node heights as mean heights. The consensus tree was further visualized using FigTree v1.6 (http://tree.bio.ed.ac.uk/software/figtree/, accessed on 26 November 2021), and divergence time bars were obtained automatically from the output using the 95% highest posterior density (HPD) of the ages for each node.

## 3. Results

### 3.1. Mitochondrial Genome Structure and Composition

In total, for two samples of *D. murina*, we obtained 37,517,290 and 16,452,636 raw pair-end reads of 75 bp in length. The average quality level was 36 on the Phred 33 scale. After filtering and cleaning from adapters, the total numbers of reads were 35,624,387 and 15,046,431, respectively, and of the same quality value. 

The average coverage of the reference mitochondrial genome (a consensus of six *Tupaia* mitochondrial genomes) was 79.5% (13,361 of 16,807 bp), and the mean coverage depth comprised ~180× in the case for the *P. lowii* raw-reads alignment. 

**Figure 2:**
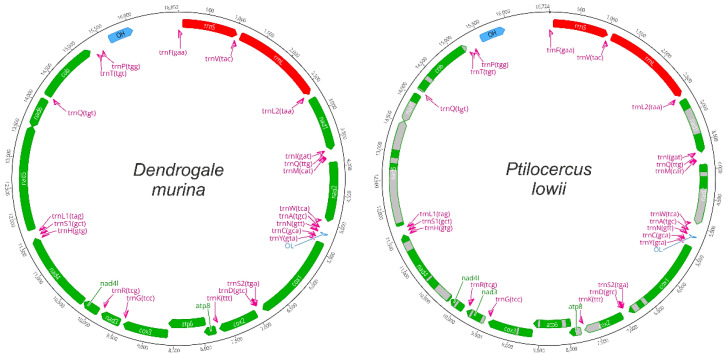
Map of the mitochondrial genomes of *Dendrogale murina* and *Ptilocercus lowii*. Green pointed bands mark annotations of protein-coding genes (CDs); rRNAs are marked in red, tRNAs in violet, and OH and OL (origins of heavy and light strand replication, respectively) colored with blue. Non-sequenced areas of *P. lowii* (SRR2577514, SRR6053052, and *CYTB* MK111982) mapped to *Tupaia* consensus sequence are marked in grey. Map of mitochondrial genomes was visualized in Geneious Prime 2019.1 and edited manually in CorelDRAW X8.

The complete mitochondrial genome of the northern smooth-tailed treeshrew *D. murina* was assembled and annotated in the current study. Data were submitted to the NCBI Nucleotide database with the accession numbers OP006204 and OP006205. It is a closed circular molecule of 16,844–16,850 bp in length and contains 37 genes—the typical set of 13 protein-coding genes (PCGs), 2 ribosomal RNA (rRNAs) genes (rrnL and rrnS), 22 transfer RNA genes (tRNAs), and a putative control region (Figure 2). The percentage of GC pairs is about 39.4%. The nucleotide composition is A, C, G, and T of approximately 33.5%, 25.5%, 13.9%, and 27.1%, respectively. The AT skew and GC skew values composed 0.11 and −0.29–−0.30, respectively, in the two assembled mitochondrial genomes of *D. murina*. Nine genes (*ND6* and eight tRNAs) were oriented in the reverse direction, whereas the others were transcribed in the forward direction. A total of 11 overlapping regions with a total length of 71 bp were identified in the *D. murina* mitochondrial genomes. The longest overlap of 43 bp in length is located between ATP synthase F0 subunits *ATP8* and *ATP6*.

The initial codons for 13 PCGs of *D. murina* were the canonical putative start codons (ATG for *ND1*, *COX1*, *COX2*, *ATP8*, *ATP6*, *COX3*, *ND4L*, *ND4*, *ND6*, and *CYTB* and ATT for *ND2*, *ND3*, and *ND5*). The typical termination codon (TAA or TAG) occurs in nine PCGs (*ND1*, *ND2*, *COX2*, *ATP8*, *ATP6*, *COX3*, *ND4L*, *ND5*, and *CYTB*); *COX1* and *ND6* terminated with AGA, *ND3*, and *ND4*; and TAA stop codon is completed by the addition of 3’ A residues to the mRNA. Detailed information about the completeness and lengths of 13 mitochondrial protein-coding genes of *D. murina* and *P. lowii* is given in Table 1.

In protein-coding genes, all the amino acids, except Tyr, are varied in codons. Amino acids Leu and Ser turned out to be the most diverse, with the frequency of CTA (31.4%), CTC (12.9%), CTG (7.8%), CTT (15.0%), TTA (25.3%), and TTG (7.5%) for Leu and AGC (18.3%), AGT (12.1%), TCA (26.6%), TCC (17.0%), TCG (4.5%), and TCT (21.5%) for Ser.

For the *P. lowii*, we succeeded in assembling a fragmented mitochondrial genome (Figure 2 and Table 1). Two ribosomal RNA genes *12S* and *16S* were complete. From the protein-coding genes, we assembled a total length of 6856 bp (approximately 61% of 13 PCGs’ length when mapping to a *Tupaia* consensus sequence as a reference). The best assembled were *COX1*, *COX3*, and *ND4L*, as they have collected more than 90% of the protein-coding-gene length. The assemblage of genes *ND5* and *ND6* was the worst of all, for which the length was 16% and 27% of the length of the whole gene, respectively. For the remaining protein-coding genes, 39 to 86% of the length was assembled. The partial mitochondrial genome of *P. lowii* was uploaded to GitHub: https://github.com/ZaTaxon/Ptilocercus_lowii.

### 3.2. Phylogenetic Reconstruction and Divergence Time Estimation

**Figure 3:**
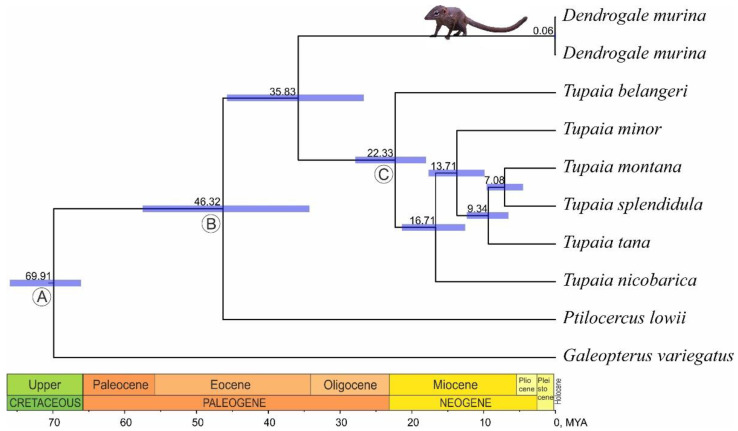
Bayesian phylogenetic reconstruction of Scandentia inferred from mitochondrial genomes. Posterior probabilities are 1.0 at each node. The divergence times (in MYA) were estimated by three calibration points (A, B, and C). Node labels represent mean node ages, and blue bars represent 95% highest posterior density (HPD) around mean estimates of divergence times. Tree topology and node bars were visualized in FigTree v1.6 and edited manually in CorelDRAW X8.

Bayesian inference (BI) and maximum-likelihood (ML) analyses for the order Scandentia based on mitochondrial genomes showed the absolutely resolved phylogenetic tree with 1.0 Bayesian posterior probabilities and 100% ML bootstrap support values for all nodes. Phylogenetic reconstruction showed the earlier derivation of *Ptilocercus* and its sister position to the rest of the group uniting *Dendrogale* and *Tupaia* genera clades (Figure 3). In turn within the latter, all studied *Tupaia* species form the monophyletic group sister to the *Dendrogale* clade. 

The separation time of the *Dendrogale* genus is estimated as an average of 35.8 MYA and 95% HPD of 45.76-26.72 MYA. *P. lowii* separation is about 46.3 MYA (95% HPD of 57.53-34.28 MYA). *Tupaia* first divergence is estimated as an average 22.3 MYA (95% HPD of 27.89-18.03 MYA) (Table 2).

## 4. Discussion

### 4.1. New Mitochondrial Genomes Assembled

In the current study, we, for the first time, sequenced and de novo assembled the complete mitochondrial genome of the northern smooth-tailed treeshrew *D. murina*. In addition, we partially assembled the mitochondrial genome of the most primitive, basal representative of the order, the pen-tailed treeshrew *P. lowii*, from the SRA data available in the NCBI SRA database. The gene order and organization of the mitochondrial genome of *D. murina*, along with mitochondrial genomes of other representatives of the family *Tupaiidae*, showed a structure typical for other vertebrates [42]. The nucleotide composition, AT and GC skew values of two *D. murina* specimens, are highly similar. Mitochondrial genomes of *D. murina* are relatively GC-poor (39.4%) compared with *Tupaia* mitochondrial genomes (where it was above 40%).

### 4.2. The Phylogenetic Relationships of Genera within the Order and Time Estimates 

We present the first phylogenetic reconstruction for the order Scandentia, including two basal genera *Dendrogale* and *Ptilocercus*, based on mitochondrial genomes.

Bayesian inference analysis showed the robustly supported phylogenetic tree with a conventional taxon pattern, revealed previously using individual molecular markers [11]. Thus, the mitochondrial genome-based phylogeny finally resolved the issue on the position of *Dendrogale* among the treeshrews, placing the *Dendrogale* as sister to the rest of the Tupaiidae. Among the hypotheses based on the morphological evidence, complete mitochondrial genome analysis supports the hypotheses of Luckett [20] and Olson et al. [10] and disagrees with the hypotheses proposed by Butler [18] and Steele [19]. The topology obtained on the base of mitochondrial genomes is also in agreement with one received in the analysis of a single *12S* rRNA gene by Olson et al. [21]. The family Ptilocercidae, which is represented with the only species, *P. lowii*, holds the sister position to *D. murina* and six analyzed species of the *Tupaia* genus (Tupaiidae family) within the order Scandentia (Figure 3). The latter arrangement is also in good agreement with phylogenetic hypotheses based both on morphological and molecular evidence [10,20,21]. Therefore, obtained results in a given case demonstrate a very good consistency in phylogenetic inference between molecular and morphological data in the case where morphological characters are thoroughly treated. A clear example of this is demonstrated in the results of three independent morphological studies of Scandentia [18,19,20]. The detailed study of quantitative and qualitative variations of exclusively dental features [19] both in a distance-based (UPGMA) analysis [19] and in further reanalysis of the same data set under the criterion of maximum parsimony [10] failed to provide phylogenetic resolution, and *Dendrogale* was placed within the genus *Tupaia*. The other study also considered exclusively dental characters [18] but, with greater attention to evaluation of character states, resulted in an equally parsimonious hypothesis of the position of *Dendrogale* either as nested within Tupaiidae or its basal-most member. Finally, the integrative analysis of dental, cranial, postcranial, and soft anatomical features unambiguously proposed phylogeny, supporting a sister relationship between *Dendrogale* and the remaining tupaiine genera [20], the one supported here by mitochondrial genome data. This case highlights the significant role of rigorous morphological studies in phylogenetics systematics. However, it should be underlined that such a good consistency in topology was achieved only with the long branches of the tree leading to taxa of the generic rank. We speculate that in the case of long evolutionary history, such consistency is not surprising, and it should be remembered here that Scandentia are often considered to be “living fossils”. However, in the case of fast explosive evolution, the unresolved phylogenies representing hard polytomies are more often and discordance between morphological and molecular trees, which is commonplace.

The branching order within the Scandentia is stable, but the dates of individual nodes differ depending on the data included in the analysis and the fossil calibrations used. The separation time of the *Dendrogale* genus is estimated as Late Eocene with an average of 35.8 MYA. Due to the use of a younger calibration point at the root [38,39] and the addition of a calibration point to the node B, the common ancestor of *Tupaia*, *Dendrogale*, and *Ptilocercus* genera, we observe an increase in the age of basal nodes within the genus *Tupaia* compared to the results of Kundu et al. [26]. Our result of dating the divergence of *Dendrogale* and *Tupaia* genera is slightly older than shown in Roberts et al. [11], who dated this split approximately to the Eocene–Oligocene boundary based on the analysis of mitochondrial ribosomal genes *12S*, tRNA-Val, and *16S*. The estimated separation time of *Ptilocercus*, on the contrary, is younger—about 46 MYA compared to the estimate of about 60 MYA made by Roberts et al. [11].

The mitochondrial genome sequencing data of the two earliest diverged genera within order Scandentia presented here may serve as an essential genetic resource for conservation purposes and for future studies of adaptive evolution of this ancient group. To better understand the overall phylogeny of this group, additional mitogenome sequences from genera *Anathana* and *Urogale* should be analyzed. However, further detailing of evolutionary history at the species level and beyond will necessarily require nuclear genes and transcriptomic data to provide better phylogenetic resolution at the tip nodes.

## 5. Conclusions

The complete mitochondrial genome of the northern smooth-tailed treeshrew *D. murina* was sequenced for the first time, and the partial mitochondrial genome of *P. lowii* was assembled from previously published SRA data. Phylogenetic analysis of Scandentia mitochondrial genomes showed a consistency with results of morphological studies, thus highlighting the significant role of rigorous morphological studies in phylogenetics systematics. The whole mitochondrial genome sequencing of *D. murina* is essential as a genetic resource for conservation purposes and for future studies of adaptive evolution within the Scandentia. For the final analysis of the phylogeny of the order Scandentia at the generic level, mitogenomic data for two monotypic genera, *Anathana* and *Urogale* within the Family Tupaiidae, are still lacking. For species-level phylogeny and taxonomy, nuclear genes and transcriptomic data should be used to provide better resolution.

## Figures and Tables

**Table 1:** List of the 13 protein-coding genes in the mitochondrial genomes of *Dendrogale murina* and *Ptilocercus lowii*.

Gene	Start	Stop	Length	Direction	fcd	scd	Completeness	Absent Fragments, aa
***Dendrogale murina* OP006204**								
*ND1*	2754	3710	957	forward	ATG	TAG	complete	-
*ND2*	3919	4962	1044	forward	ATT	TAG	complete	-
*COX1*	5344	6891	1548	forward	ATG	AGA	complete	-
*COX2*	7028	7711	684	forward	ATG	TAG	complete	-
*ATP8*	7777	7980	204	forward	ATG	TAA	complete	-
*ATP6*	7938	8618	681	forward	ATG	TAA	complete	-
*COX3*	8618	9403	786	forward	ATG	TAA	complete	-
*ND3*	9472	9818	347	forward	ATT	TAA*	complete	-
*ND4L*	9886	10,182	297	forward	ATG	TAA	complete	-
*ND4*	10,176	11,553	1378	forward	ATG	TAA*	complete	-
*ND5*	11,751	13,562	1812	forward	ATT	TAA	complete	-
*ND6*	14,079	13,558	522	reverse	ATG	AGA	complete	-
*CYTB*	14,152	15,291	1140	forward	ATG	TAG	complete	-
***Dendrogale murina* OP006205**								
*ND1*	2754	3710	957	forward	ATG	TAG	complete	-
*ND2*	3919	4962	1044	forward	ATT	TAG	complete	-
*COX1*	5344	6891	1548	forward	ATG	AGA	complete	-
*COX2*	7028	7711	684	forward	ATG	TAG	complete	-
*ATP8*	7777	7980	204	forward	ATG	TAA	complete	-
*ATP6*	7938	8618	681	forward	ATG	TAA	complete	-
*COX3*	8618	9403	786	forward	ATG	TAA	complete	-
*ND3*	9472	9818	347	forward	ATT	TAA*	complete	-
*ND4L*	9886	10,182	297	forward	ATG	TAA	complete	-
*ND4*	10,176	11,553	1378	forward	ATG	TAA*	complete	-
*ND5*	11,752	13,563	1812	forward	ATT	TAA	complete	-
*ND6*	14,080	13,559	522	reverse	ATG	AGA	complete	-
*CYTB*	14,153	15,292	1140	forward	ATG	TAG	complete	-
***Ptilocercus lowii***								
*ND1*	2755	3711	957	forward	ATG	TAG	partial	2914–3418;
*ND2*	3916	4957	1042	forward	ATA	-	partial	4074–4134; 4321–4957
*COX1*	5348	6889	1542	forward	ATG	TAA	partial	6565–6619; 6751–6836
*COX2*	7032	7715	684	forward	ATG	-	partial	7421–7715
*ATP8*	7783	7989	207	forward	-	TAG	partial	7783–7896
*ATP6*	7944	8624	681	forward	ATG	TAA	partial	8030–8106; 8496–8516
*COX3*	8624	9408	785	forward	ATG	TAA*	partial	8880–8898
*ND3*	9477	9823	347	forward	-	TAA*	partial	9477–9530; 9711–9757
*ND4L*	9897	10,151	255	forward	ATG	TAA	partial	10,023–10,043
*ND4*	10,145	11,521	1377	forward	ATG	TAA	partial	10,235–10,477; 10,640–10,658; 11,275–11,459
*ND5*	11,722	13,534	1813	forward	ATT	-	partial	11,791–12,704; 12,805–12,918; 13,031–13,534
*ND6*	14,057	13,537	521	reverse	ATG	-	partial	13,537–13,918;
*CYTB*	14,131	15,270	1140	forward	-	-	partial	14,131–14,148; 14,869–14,947; 15,203–15,270

Start—the first position along α strand; Stop—the last position along α strand; Length—the size of the sequence; fcd—first codon; scd—stop codon. For partial gene sequences, missing fragments are indicated in the last column. Cases where TAA stop codon is completed by the addition of 3’ A residues to the mRNA are marked with an asterisk.

**Table 2:** Divergence time estimates for the major lineages within Scandentia.

Taxon Separated	Mean	95% HPD
* Ptilocercus lowii *	46.32	57.53–34.28
* Dendrogale murina *	35.83	45.76–26.72
* Tupaia belangeri *	22.33	27.89–18.03
* Tupaia nicobarica *	16.71	21.4–12.59
* Tupaia minor *	13.71	17.67–9.89
* Tupaia tana *	9.34	12.35–6.54
* Tupaia montana / T. splendidula *	7.08	9.58–4.52

Mean node ages 95% highest posterior density intervals are given in million years ago.

## Data Availability

Mitochondrial genomes of *D. murina* were submitted to the NCBI GenBank database under the accession numbers OP006204 and OP006205. The partial mitochondrial genome of *P. lowii* was uploaded to github: https://github.com/ZaTaxon/Ptilocercus_lowii.
